# PSYCHOLOGICAL FACTORS ASSOCIATED WITH AMBULATION PERFORMANCE AFTER HIP FRACTURE AMONG HOME-DWELLING OLDER ADULTS

**DOI:** 10.1093/geroni/igz038.1566

**Published:** 2019-11-08

**Authors:** Richard H Fortinsky

**Affiliations:** University of Connecticut School of Medicine, Farmington, Connecticut, United States

## Abstract

Many older adults fail to resume optimal community living after hip fracture due to sustained limitations in ambulation capacity, yet reasons remain poorly understood. Roles of psychological factors in affecting ambulation performance post-hip fracture remain particularly understudied; depression has been associated with poorer self-reported functional status, and little is known about self-perceived balance confidence, resilience, and optimism. This presentation reports associations between each psychological factor, measured at CAP baseline, and gait speed and walking endurance, measured at baseline and 16 weeks later. In the CAP cohort (N=210), baseline mean/sd 4-meter gait speed (gs), 50-foot walk gs, and 6-minute walk distance were: 0.60/0.19 meters per second (mps); 0.67/0.20 mps; and 186.9/55.4 meters, respectively. In multivariate models, balance confidence was positively associated with all baseline ambulation measures (p<0.001 in all models), and resilience was positively associated with all 16-week follow-up ambulation measures (p>0.05 in all models). Implications of results will be discussed.

